# Ponderal Status, Eating and Lifestyle Habits in Rural School Children: A Pilot Survey of the SBAM-ONFOODS Cohort Study

**DOI:** 10.3390/nu18111756

**Published:** 2026-05-29

**Authors:** Myriam Galfo, Laura D’Addezio, Romana Roccaldo, Laura Censi

**Affiliations:** Consiglio per la Ricerca in Agricoltura e l’Analisi dell’Economia Agraria (CREA), Centro di Ricerca Alimenti e Nutrizione (Council for Agricultural Research and Economics, Research Centre for Food and Nutrition), Via Ardeatina 546, 00178 Rome, Italy; myriam.galfo@crea.gov.it (M.G.); laura.daddezio@crea.gov.it (L.D.); romana.roccaldo@crea.gov.it (R.R.)

**Keywords:** children, ponderal status, food habits, lifestyle

## Abstract

**Background/Objectives**: The rising prevalence of overweight and obesity reflects a trend towards worsening eating habits and reduced physical activity, with significant implications for public health and life expectancy. This pilot survey aimed to evaluate weight status, food habits and lifestyle in primary school children from under-researched rural areas of the Lazio region, Italy. **Methods**: A total of 182 children aged 6–10 years from the municipalities of Monte Romano and Tolfa were enrolled. Body Mass Index (BMI), calculated from measured weight and height, was classified by IOTF and WHO definitions. Food habits, socioeconomic factors and lifestyle were evaluated by a standard questionnaire, and adherence to the Mediterranean diet (MD) was assessed by the KIDMED test. **Results**: Data showed a high prevalence of overweight, including obesity (33.3%), based on IOTF criteria, with slightly higher rates in boys than girls (38.3% vs. 26.9%). According to the WHO definition, the rate of overweight/obesity was 39.5% with significant differences between males and females (46% vs. 30.8%). Only 19.9% of children had high adherence to MD (67.3% moderate, 12.9% low), and 29.5% of the sample did not eat breakfast every day. In addition, 25.4% met international physical activity recommendations, while around 40% exceeded the recommended screen time on weekend days, and 19.3% did not sleep the recommended hours. **Conclusions**: Preliminary data show high rates of excess weight, poor diet quality and unhealthy lifestyles. Extending the sampling to other municipalities in the study area will help validate these findings and provide deeper insights to inform targeted intervention strategies.

## 1. Introduction

The global rise in overweight and obesity prevalence reflects a trend towards inadequate diets and low physical activity levels across various population groups. This tendency could result in an increase in chronic diseases in the future, negatively affecting both quality of life and life expectancy [1]. Overweight and obesity in childhood represent one of the main public health challenges for the WHO European Region [2]. Children with obesity are at greater risk of developing early comorbidities and complications, including metabolic disorders, hypertension, respiratory problems, sleep disorders, orthopaedic problems and psychological distress, such as low self-esteem and social stigma. Furthermore, obese children are more likely to become obese adults and to develop non-communicable diseases (NCDs) both at an earlier age [3] and later in life [4,5]. Despite a slight improvement in recent years, Italy remains one of the European countries with the highest rates of overweight and obese school-age children, along with other Southern European countries, such as Cyprus, Greece and Malta, as highlighted by the WHO European Childhood Obesity Surveillance Initiative (COSI) survey [6]. Specifically, the most recent survey conducted by the national surveillance system OKkio alla SALUTE in 2023, reported excess weight—overweight, including obesity—in approximately three out of ten primary school children in Italy [7], based on the reference curves of the International Obesity Task Force [8].

Furthermore, unhealthy eating habits, including low fruit and vegetable consumption, are widespread among European children. The percentage of children who consume fruit and vegetables daily varies greatly across European countries, with Italian children showing values close to the overall average [9].

The Mediterranean diet (MD) is mainly characterised by a high intake of plant-based foods—such as fruit, vegetables, legumes, nuts and whole grains—extra virgin olive oil as the main source of fat, low consumption of red meat and moderate intake of dairy products, poultry, eggs and fish [10]. The MD, recognised by UNESCO as an Intangible Cultural Heritage of Humanity in 2010 [11], is based on traditional, local and environmentally sustainable food practices that support biodiversity and cultural heritage. It also includes lifestyle elements such as moderation, seasonal consumption, conviviality, culinary traditions, intergenerational knowledge, time dedicated to meals, adequate physical activity and rest [10]. Although MD has well-documented beneficial effects on a range of health outcomes, there is also growing evidence that this traditional healthy eating pattern is being increasingly abandoned in Mediterranean countries, especially among children and adolescents [12,13]. The distribution of KIDMED categories—the most widely used method for assessing adherence to the MD in children, as proposed by Serra-Majem et al. [14]—has revealed substantial variability in MD adherence: poor adherence ranges from 1.6% in Spanish children to 62.8% in Greek adolescents; moderate adherence from 28.0% in Greek adolescents to 73.8% in rural Italian adolescents; and high adherence from 4.3% in Greek children (10–12 years) to 53.9% in Spanish children [15]. Studies conducted in Italy have also highlighted a tendency among school-age children to abandon the MD, mainly manifested by a reduced consumption of vegetables, fruit and legumes [16,17,18].

Thus, in light of the global shift away from traditional dietary patterns towards diets characterised by low consumption of plant-based foods and high intake of foods rich in saturated fats, sugars and salt (associated with an increased risk of non-communicable diseases such as cardiovascular disease, type 2 diabetes and certain cancers [19,20,21]), it is essential to implement targeted strategies to improve nutritional status, diet quality and lifestyle at critical stages of life [22]. Urbanisation has been correlated with changes in lifestyle and dietary patterns and has been weakly associated with reduced adherence to the MD in children. Limited evidence from a small number of studies suggests that residing in rural areas may be linked to more traditional dietary behaviours [15]. Assessing MD adherence and its key determinants is essential to guide strategies that promote healthy eating habits, particularly during childhood and adolescence [15].

It is worth noting that innate preferences and genetics influence taste in early childhood, while early experiences also shape long-term taste preferences and eating behaviours that tend to persist into adulthood; moreover, taste preferences are crucial factors in determining food intake [23,24]. Improving eating habits, especially at this stage of life, can promote health and reduce the risk of diseases later in life [25].

Engaging in adequate physical activity is also essential for lifelong well-being, with several benefits for both physical and mental health. It also plays a crucial role in supporting energy balance and maintaining a healthy body weight. Establishing healthy movement habits during childhood contributes to the development of physical activity behaviours that carry through adolescence and adulthood. In children and adolescents, physical activity improves physical fitness, cardiometabolic health and motor and cognitive development, promotes bone health and healthy growth and development and reduces adiposity. The WHO recommends that children and adolescents aged 5 to 17 years engage in at least 60 min of moderate-to-vigorous physical activity per day, primarily aerobics, across the week. Additionally, vigorous-intensity activities, including those that strengthen muscles and bones, should be included at least three times a week [26]. Physical activity may take place in educational, home or community settings, through recreational and leisure activities, physical education (e.g., games, sports or structured exercise), active transportation (e.g., walking or cycling) and even household chores.

Physical inactivity is considered one of the leading causes of global mortality [26]. Although insufficient physical activity and sedentary behaviour may appear similar, they represent conceptually distinct constructs [27]. Insufficient physical activity refers to not achieving the levels of physical activity recommended by current guidelines [26], whereas sedentary behaviour encompasses any waking behaviour characterised by low energy expenditure while sitting, reclining or lying [28]. The WHO Global Action Plan on Physical Activity 2018–2030 aims to achieve a 15% reduction in physical inactivity by 2030 [29]. In Italy, the promotion of physical activity is recognised as a public health priority, in line with the objectives of the WHO Action Plan and European Union strategies [30,31].

In children and adolescents, higher levels of sedentary behaviour are associated with adverse health outcomes; therefore, the World Health Organization recommends limiting sedentary time, particularly recreational screen use [26].

Notably, screen time (i.e., the time children spend watching television or using electronic devices with screens) contributes to increased sedentary behaviour, which has been shown to correlate with unhealthy dietary habits [32]. Moreover, excessive screen time not only reduces opportunities for physical activity but may also negatively influence eating habits, partly due to likely greater exposure to advertising for unhealthy foods. This increased exposure can lead to higher food intake, particularly among overweight or obese children [33].

Although participation in sport appears to be trending upward, particularly among young people, overall physical activity levels in Italy remain limited. Among people aged 3 years and older, sports participation varies significantly across Italy: the highest rates are recorded in the Northern and Central regions, where roughly four in ten people engage in sports, whereas the South and the islands show the lowest levels, with fewer than three in ten participants. Differences also emerge when considering the size of municipalities: participation is lowest in small municipalities with up to 2000 inhabitants (29.7%) and highest in metropolitan centres (42.7%) and in municipalities bordering metropolitan areas (40.1%) [34]. Moreover, the proportion of people aged 3 years and older who do not engage in any sports or physical activity remains high in Italy and increases progressively from the North through the Centre to the South, where nearly half of the population (47.5%) is inactive [35].

Based on these considerations, it is essential to gain a deeper understanding of the nutritional status, lifestyle and their modifiable determinants among school children, especially those living in under-researched areas of Italy, such as small municipalities that might not have been included in regional or nationwide programs due to their limited size.

The aim of the SBAM study is to assess the weight status, eating habits and physical activity of primary school children (ages 6 to 10) in little-studied rural areas of Italy, and to interpret these findings in the context of available national data from the OKkio alla SALUTE surveillance system. This assessment seeks to identify needs and gather the knowledge necessary for developing effective, targeted actions to improve the diet quality and lifestyle of children in these communities.

## 2. Materials and Methods

### 2.1. Study Design

This study is part of a national project on the topic of “Models for sustainable nutrition” entitled “ON Foods—Research and innovation network on food and nutrition Sustainability, Safety and Security—Working ON Foods”, selected within one of the fourteen partnerships envisaged by the National Recovery and Resilience Plan (NRRP). ON Foods is a partnership of 26 public and private organisations, including universities, research centres and companies, leaders in scientific research and sustainable innovation of food systems. In particular, the SBAM study is developed within Spoke 5 “Lifelong Nutrition”, which, among its objectives, aims to create a map of the nutritional status of different population groups in different Italian geographical areas, including urban and rural, economically advantaged and peripheral.

The study is an observational cross-sectional study. The setting is primary school. The rural municipalities of Tolfa and Monte Romano in the Lazio region were selected for this pilot study. According to data from the Italian National Institute of Statistics (ISTAT), the official source of demographic statistics in Italy, the resident population in 2023 was 4763 inhabitants in Tolfa and 1872 in Monte Romano [36]. Standardised methodologies were used for data collection to enable comparison with data collected in urban and rural areas across other Italian regions and with national data.

### 2.2. Sample and Data Collection

A non-probabilistic, voluntary sample was recruited. All children enrolled in primary schools in the municipalities of Monte Romano and Tolfa were invited to participate in the study through the involvement of the school principals and the school representatives or “trustees”, to whom the project and its objectives were presented. Participants were recruited from the only primary schools in each municipality, with all classes in each school involved. In total, 241 children were enrolled. Only children whose parents both provided signed consent were included in the study. The total participation rate was 74.6% (71.4% for Monte Romano and 77.7% for Tolfa), with a similar rate between sexes, for a total of 182 children included (2 children did not have anthropometric measurements).

According to data from ISTAT [36], a total of 89 children aged 6–10 years resided in Monte Romano as of 1 January 2023 and 190 in Tolfa as of 1 January 2024. The sampling fractions were 60 out of 89 (0.65) for Monte Romano and 122 out of 190 (0.64) for Tolfa. Moreover, a post hoc calculation of the minimum sample size required for estimating a proportion based on the formula n_0_ = z^2^ p(1 − p)/E^2^ subsequently adjusted for finite population (n = n_0_/[1 + (n_0_ − 1)/N]) confirmed that the sample of 182 children was sufficient to estimate the proportion of children with overweight/obesity in the 6–10-year age group, where z is the z -score corresponding to the desired confidence level (1.96 for 95% confidence), E is the margin of error (for ±5% = 0.05) and p is the estimated proportion of children with overweight/obesity assumed equal to 0.28 from previous studies. The collaboration of the teachers involved was crucial for the successful collection of data. The planning of data collection was coordinated with the school principal and the teachers to minimise disruption to teaching activities. Data collection took place in May 2023 (Monte Romano) and January 2024 (Tolfa).

To gather information on children’s eating habits, lifestyle and living environment, the “Questionario dei genitori SBAM,” completed by one of the parents, was used. The questionnaire includes a section adapted from the “Questionario dei genitori OKkio alla SALUTE 2023”. Its use for this project was authorised by the coordinator of the national surveillance system, OKkio alla SALUTE, and was intentionally adopted to ensure comparability with nationally collected data and to allow a contextualised interpretation of the findings within the Italian surveillance framework. To assess whether children’s fruit and vegetable consumption is adequate in relation to the WHO recommendation [37], the “Questionario dei genitori SBAM” includes questions on the number of both portions of fruit and vegetables on the days in which children consume these foods. A section was also added to the questionnaire for the evaluation of diet quality and adherence to the MD by the KIDMED test (Mediterranean Diet Quality Index for children and adolescents) [14]. The test includes 16 yes/no items based on the principles of the MD. Responses with a negative connotation are scored −1, and those with a positive connotation are scored +1. The overall score ranges from −4 to 12: a score of 3 or lower indicates poor adherence to the principles of the MD (low adherence), scores between 4 and 7 indicate average adherence and scores between 8 and 12 indicate high (optimal) adherence. The KIDMED scoring sheet is included as Supplementary Table S1.

### 2.3. Anthropometric Data

The children’s weight and height measurements were carried out by two trained and standardised observers following the techniques described by Lohman et al., 1988 [38]. The anthropometric measurements took place at school, in the morning, in the presence of the teacher, in a room specially designated by the school, ensuring privacy and confidentiality for each child.

The body weight of each child, fasting (or after a light breakfast), was measured to the nearest 50 g using a SECA 872™ electronic scale (Hamburg, Germany). To encourage the participation of schools, families and children in the study, and in accordance with the protocol of the Italian national surveillance system, OKkio alla SALUTE [39], a validated procedure was used [40]. Following this procedure, children were measured while wearing light indoor clothing, after removing shoes, school aprons, heavy clothing (e.g., sweaters, jackets, sweatshirts), belts, items from pockets and any accessories. The child’s clothing during weight measurement was recorded on the measurement sheet using a checklist. During data processing, the measured body weight was corrected for the estimated weight of each garment worn by subtracting a standard weight value, estimated by measuring several samples of similar garments in sizes suitable to the age group examined.

Height was measured to the nearest 0.1 cm using the SECA 214™ stadiometer (Hamburg, Germany). The subject stood without shoes, with weight evenly distributed on both feet and the head oriented in the Frankfurt plane. The back of the head, buttocks and heels were in contact with the vertical board of the stadiometer. To evaluate the weight status of the children, Body Mass Index (BMI) was calculated for each child by dividing their weight in kilograms by the square of their height in meters (kg/m^2^). The resulting BMI values were then compared with the international reference curves specific to sex and age, including those established by the International Obesity Task Force [8] and the World Health Organization [41,42], two of the most widely used systems, to allow comparability with other studies.

### 2.4. Statistical Analysis

Descriptive analyses are presented as means ± standard deviations for quantitative variables and as percentages for categorical variables. Chi-square test and T-tests were used to test for significant proportions and mean differences, respectively. A multivariable binary logistic regression model was employed to examine the association between selected food habits and lifestyle factors (independent variables) and high adherence to the Mediterranean diet (dependent variable, coded as 1 = high adherence, 0 = low/moderate adherence). All variables of interest were included simultaneously using the enter method. Analyses were conducted with unadjusted and adjusted covariates. For all the tests, *p*-values < 0.05 were considered statistically significant. All statistical analyses were performed using the computer software IBM SPSS Statistics, version 250.0 (SPSS Inc., Chicago, IL, USA).

## 3. Results

Anthropometric and socio-demographic characteristics of the study sample are presented in Table 1. The percentage of boys in the sample exceeded that of girls (57.1% vs. 42.9%, respectively). Participants’ characteristics were as follows (mean ± standard deviation): age 8.5 ± 1.5, weight 31.5 ± 9.2 kg, height 131.9 ± 10.1 cm, BMI 17.8 ± 3.3 kg/m^2^. The prevalence of overweight, including obesity, was 33.3%, according to the IOTF definition, with higher values in boys than in girls (38.3% vs. 26.9%), although this difference was not significant. According to the WHO definition, the rate of overweight, including obesity, was 39.5%, with significant differences between males and females (respectively, 46% vs. 30.8%; *p* = 0.0174). Most mothers in the sample had a high school diploma (44.6%) or a university degree/postgraduate degree (33.7%), while 60% of fathers had a high school diploma, and 28.3% had less than middle school education. In the overall sample, a significant inverse association was observed between maternal education and the prevalence of overweight/obesity when applying the IOTF criteria: the proportion of children with overweight or obesity was significantly lower among those whose mothers had a university or postgraduate degree (21.4%) compared with those whose mothers had a high school education or lower (38%). Using WHO criteria, a similar pattern emerged, with a lower prevalence of overweight/obesity among children of mothers with higher educational attainment (32.1% vs. 42.6%); however, the difference was not statistically significant.

Table 2 shows food habits and lifestyle factors among the study sample. About 13% of participants had a low MD adherence, 29.5% did not consume breakfast daily, only 42.6% ate fruit once or more a day, as many as 73.3% did not eat vegetables every day, while 30.2% consumed legumes less than once a week. As regards the consumption of sugary drinks, sweets and various snacks, 68.2% never or rarely consumed sugary drinks, approximately 18% ate sweet snacks one or more times a day, while 53.7% never or rarely consumed salty snacks. Concerning the lifestyle of the sample, 80.7% slept 9 or more hours as recommended [43], 33.5% did non-structured physical activity 5/7 days a week and only 2.3% played sports 5/7 days a week. In addition, 92% spent more than 2 h on screen time in contrast to international recommendations, which advise limiting the amount of time spent being sedentary, particularly recreational screen time [26], 59.3% had a television in their bedroom and only 13.1% walked to and from school.

**Figure 1:**
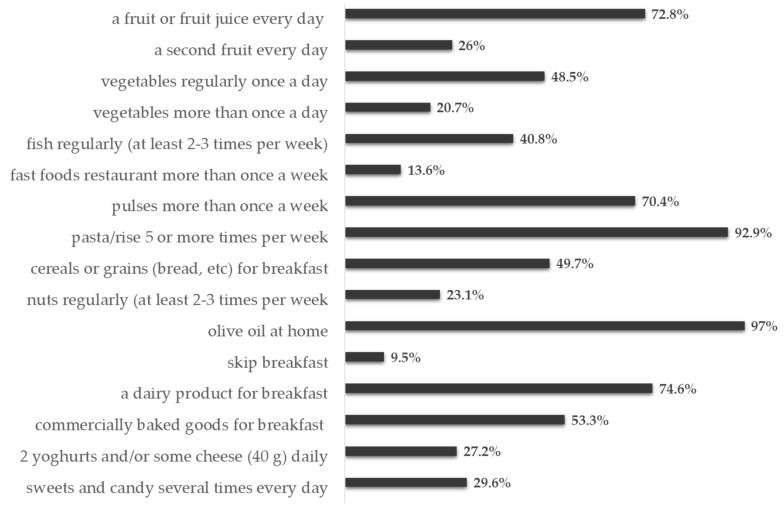
KIDMED test components.

Figure 1 describes the distributions for the specific components of the KIDMED index. The study found that 72.8% of the children habitually consumed one fruit or fruit juice every day, and 26.0% ate a second fruit every day. Concerning the consumption of raw or cooked vegetables, 48.5% ate them regularly once a day, and 20.7% ate them more than once a day. Regarding breakfast, 9.5% skipped it, 74.6% ate a dairy product for breakfast (yogurt, milk, etc.), 53.3% ate commercially baked goods or pastries and 49.7% ate cereals, bread and rusks. Finally, 29.6% ate sweets and candies several times a day. No statistically significant differences in KIDMED scores were observed across maternal education levels.

**Figure 2:**
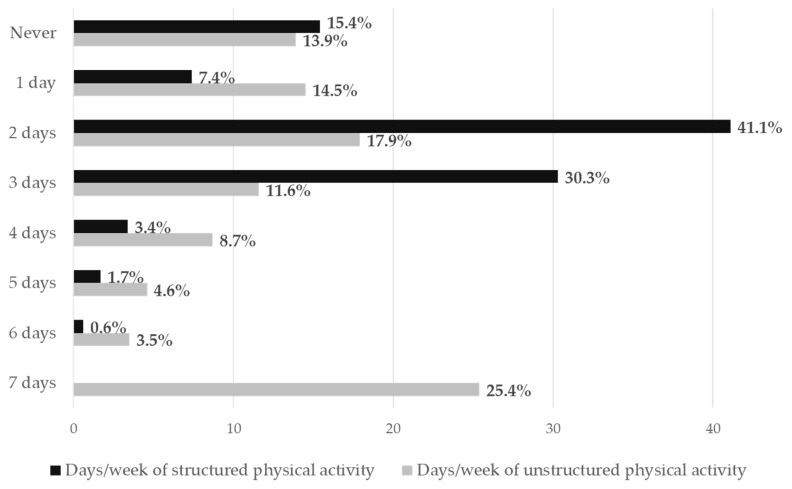
Days a week of total physical activity for at least 1 h/day.

Figure 2 shows the number of days per week that children engaged in structured physical activity (sports) and unstructured physical activity. The results showed that 41.1% of the children participated in at least 1 h of structured sports activity outside school hours for 2 days a week, 1.7% for 5 days a week, while 15.4% did not engage in any sports activity. According to parents’ reports, 25.4% of the children involved in the survey engaged in at least 1 h of physical activity every day in line with WHO recommendations [26]. Additionally, 11.6% engaged in physical activity for 3 days a week, 8.7% for 4 days a week, while approximately 14% never engaged in any physical activity.

**Figure 3:**
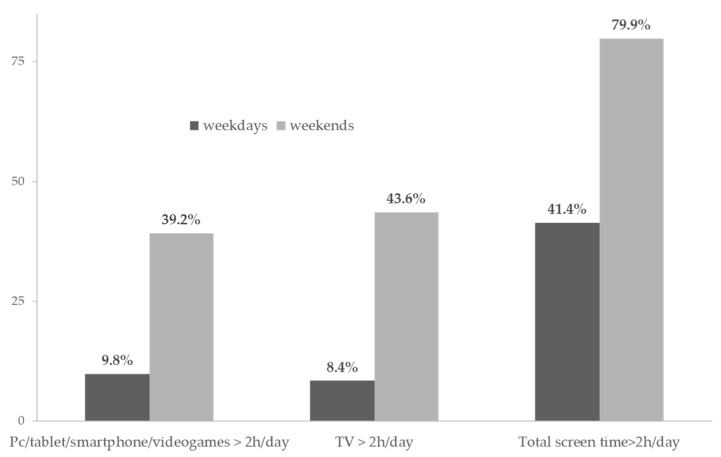
Screen time during weekdays and weekends.

Figure 3 shows children’s screen time. The percentage of children who spent more than 2 h per day in front of the TV increased during the weekend (43.6%) compared with weekdays (8.4%). The tendency to spend more than 2 h per day in front of the computer/tablet/smartphone or playing videogames was also higher on weekends (39.2%) compared with weekdays (9.8%). In total, 41.4% of children spent more than 2 h on screen time on weekdays and about 80% on weekends.

Crude and adjusted odds ratios for food habits and lifestyle factors associated with high adherence to the MD, obtained from a multivariable binary logistic regression model, are shown in Table 3. The model showed a moderate overall fit. The Omnibus test indicated that the model was statistically significant (Chi-square test, *p* = 0.002), suggesting that the predictors jointly improve the model compared with the null model. Pseudo-R^2^ values (Cox & Snell R^2^ = 0.20; Nagelkerke R^2^ = 0.32) indicate a moderate explanatory power. The Hosmer–Lemeshow test was not significant (*p* = 0.38), suggesting no evidence of poor fit. Ponderal status (WHO criteria), breakfast habit and mother’s education showed a significant association with high MD adherence, whereas ponderal status based on IOFT criteria, sugary drinks consumption, fruit consumption, vegetable consumption, sweet snack, legume consumption, screen time, TV in bedroom, non-structured physical activity and sleep time had no significant association. The results show that compared with children with overweight or obesity, those who were underweight or of normal weight exhibited 83% lower odds (OR = 0.17) of high adherence to the Mediterranean diet. In contrast, children who consumed breakfast every day had 5.7 times higher odds of high adherence to the MD compared with those who did not eat breakfast daily. Furthermore, children whose mothers had a low–medium level of education exhibited 63% lower odds (OR = 0.37) of high adherence to the MD compared with children whose mothers had a university or postgraduate education.

## 4. Discussion

This pilot survey assessed ponderal status, based on measured weight and height, as well as dietary habits and lifestyle among primary school children in two rural municipalities of Lazio in Italy. The findings aim to strengthen the evidence base needed to inform targeted health promotion strategies in these under-researched communities. The study area lies within the rural territory of northern Lazio, historically known as Tuscia, which is characterised by low population density, agricultural landscapes and shared socio-cultural and environmental features [44].

### 4.1. Overweight and Obesity

The research findings showed a high prevalence of excess body weight in this rural area, with just over three out of ten children classified as overweight or obese according to the IOTF definition [8], and slightly higher rates among boys than girls. These overweight (including obesity) prevalence rates slightly exceed those reported at both the regional level in Lazio, where the combined prevalence is 28.8%, with 19.6% classified as overweight and 9.2% as obese, and at the national level, where the prevalence is 28.8% overall—19.0% for overweight and 9.8% obesity—based on data from the 2023 OKkio alla SALUTE National Surveillance System survey [7]. According to the same national survey, prevalence rates were slightly lower in girls, as observed in our sample, and higher in southern Italian regions and among families with a disadvantaged socioeconomic status. Notably, the OKkio alla SALUTE survey also reported a decreasing trend, particularly in the prevalence of overweight, over the successive survey cycles, decreasing from 23.2% in 2008–2009 to 19.0% in the most recent wave.

In our study, the prevalence of overweight and obesity varied by classification system, with higher estimates and significant sex differences observed only using WHO criteria. These discrepancies reflect the different methodological approaches and reference populations underlying the two systems: WHO is based on BMI-for-age z-scores from a reference population, whereas IOTF cut-offs are derived from pooled international datasets and linked to adult BMI thresholds [8,42]. Accordingly, WHO criteria are more sensitive, identifying a greater proportion of children due to lower cut-offs, while IOTF criteria are more conservative and specific, relying on higher thresholds and thereby reducing false-positive classifications [45,46]. As a result, prevalence estimates may differ, and subgroup differences, including those by sex, may emerge or become more pronounced, particularly for individuals near the cut-off points. For this reason, reporting both definitions has been recommended to enhance comparability across studies [47].

From a public health perspective, the use of WHO criteria may be preferable for prevention and screening, given their higher sensitivity [45], particularly in countries such as Italy, where childhood obesity remains a significant public health concern, as well as in relatively understudied rural settings. However, BMI classifications remain screening tools and should be complemented at the individual level by clinical and body composition assessments.

### 4.2. Dietary Habits

Our study also revealed widespread dietary habits that do not align with current nutritional recommendations. Only about seven out of ten children ate breakfast every day, a figure in line with the results in the Lazio region by the 2023 OKkio alla Salute survey [7]. This is a key aspect to explore in depth to help counteract childhood obesity, as a higher prevalence of obesity has been observed among children who regularly skip breakfast [48], a result also confirmed in the OKkio alla SALUTE survey [7]. Still, regarding this key meal, it is worth noting that more than half of the sample consumed commercially baked goods or pastries (typically characterised by elevated levels of refined sugars and saturated fats, alongside low dietary fibre content) rather than cereals and grains (e.g., bread and rusks). In pediatric populations, dietary patterns rich in sweets and ultra-processed foods have been associated with insulin resistance, adverse lipid profiles and increased cardiometabolic risk [49,50].

Moreover, among the children included in the study, the habit of consuming fruit and vegetables is far from meeting the recommended intake of at least five portions a day, which is advised to improve overall health and reduce the risk of certain NCDs [37]. Specifically, only 42.6% consumed fruit at least once per day, while just 26.7% ate vegetables at least once per day. This finding is consistent with the 2023 OKkio alla Salute survey, which showed that 43% of Italian children consumed fruit at least once per day, and 30.1% ate vegetables at least once per day [7]. The WHO-COSI survey [9] found that in Europe, only 43% of children aged 6–9 consume fresh fruit daily, and just 34% eat vegetables every day, with significant variation across countries. Daily fruit consumption is slightly more common among girls, while vegetable intake is similar between sexes. Children whose parents have a higher level of education are more likely to consume fruit and vegetables daily than those whose parents have lower educational levels.

Moreover, the habit of consuming legumes was not widespread among the children surveyed in the Tuscia area, where approximately 30% were found to consume them less than once a week or never, although this percentage is slightly lower than the national average of 37.0%, as reported by the 2023 OKkio alla SALUTE survey [7].

Interestingly, the results of this pilot study suggest that, even within this rural context, children’s eating patterns deviate significantly from the principles of the MD. Specifically, fewer than two out of ten children showed optimal adherence to the MD, mainly reflecting the low consumption frequency of key MD foods, such as fruits, vegetables and legumes. Nevertheless, adherence to the MD in this area of Tuscia was higher than that observed in a 2023 study on a cohort of 132 Italian children aged 2 to 17.9 years from San Giovanni Rotondo (FG), in Puglia, southern Italy, where 71.2% showed poor adherence, 26.5% moderate adherence and only 2.3% good adherence [51]. Slightly better results were reported by a survey, also conducted in Puglia, among 282 children aged 6 to 8 years attending primary schools across five small villages in the Salento Peninsula. That study reported low adherence to the MD in 27.0% of children, moderate adherence in 59.6% and high adherence in 13.5% [52]. Similar results emerged from a 2017 study in Novara, a city in northern Italy, involving primary school children, where 19.0% showed high adherence, 60.3% moderate adherence and 20.7% low adherence [16]. Moreover, a study involving 1135 adolescents aged 13 to 16 years from 13 secondary schools in Sicily (southern Italy), selected to represent both urban and rural areas, reported a generally poor overall diet quality. The findings revealed a significant departure from the MD model, particularly among adolescents living in urban environments [53]. Collectively, these findings highlight a progressive shift away from traditional Mediterranean dietary patterns, even in rural areas where such habits would typically be expected to persist.

The logistic regression analysis showed that having breakfast every day was positively associated with high adherence to the MD. This finding is consistent with previous studies. Bôto et al., in a sample of 325 Portuguese adolescents aged 15–19 from public secondary schools in Algarve, identified regular breakfast consumption as a significant predictor of higher MD adherence [54]. Similarly, Mounayar et al. reported that Lebanese adolescents who skipped breakfast showed poorer adherence to the MD [55]. Supporting evidence also comes from the HELENA study, which involved over 1800 European adolescents and found that regular breakfast intake was associated with better overall diet quality and stronger adherence to the MD, suggesting that breakfast habits may reflect broader healthy lifestyle patterns [56].

In addition to breakfast habits, our analysis also showed that lower maternal education was associated with markedly reduced likelihood of high MD adherence in children. Similar associations have been reported in Italian populations and in other Mediterranean countries, where parental education emerges as an independent predictor of better dietary quality in children [53,57].

### 4.3. Physical Activity and Sedentary Behaviours

Our survey indicated that a large proportion of children did not meet WHO recommendations for physical activity [26]. Just over four in ten engaged in at least 60 min of structured sports outside school 2 days per week, and only about three in ten did so three days per week. In addition, only one-third of children (33.5%) participated in unstructured physical activity, such as free play, for at least 60 min a day 5 to 7 days a week. Notably, more than one in ten children did not engage in structured sports (15.4%) or unstructured activity (13.9%).

The observed patterns in small rural municipalities might be partly explained by environmental, economic and social barriers, including greater distances to sports facilities, financial constraints, limited access to recreational facilities and fewer structured and community-based opportunities, all of which may contribute to low levels of physical activity [58]. Differences in access to physical activity opportunities, particularly in small rural municipalities, might be partly explained by the uneven implementation of school-based initiatives. In Italy, such initiatives, including curricular physical education and projects developed in collaboration with national institutions, have been progressively introduced but have not always been systematic or widespread and may not have consistently reached all schools and students [59], indicating potential areas for further improvement.

These findings among children living in a rural area are broadly consistent with national data. The OKkio alla Salute Surveillance System reported that 39.3% of children practiced sports 2 days per week, 21.8% 3 days per week and 31.6% engaged in movement-based free play for at least 1 h per day 5 to 7 days per week [7]. Similarly, ISTAT data indicated insufficient levels of physical activity among Italian children aged 6–10 years: 64% of the children practiced sports regularly, 4.3% occasionally, 10% engaged in some form of physical activity, while 17.7% did not practice any physical activity at all [60].

In parallel with low levels of physical activity, this survey also found that exceeding 2 h of screen time was a widespread behaviour among the children surveyed, with the proportion increasing substantially during weekends and involving nearly eight out of ten children. These results align with national data from the 2023 OKkio alla Salute Surveillance System, which reported that 45.1% of Italian children exceeded 2 h of daily screen time on weekdays, with rates rising sharply to 84.1% during weekends [7].

Across the 29 countries participating in the COSI initiative between 2022 and 2024, an average of 42% of children aged 6–9 spent more than 2 h per day watching television or using electronic devices on weekdays, with this proportion rising to 78% during weekends. Marked cross-country differences were observed, and Italy ranked among the European countries with the highest proportions of children exceeding 2 h of daily screen time [2].

No significant association was observed between screen time and adherence to the Mediterranean diet in the multivariable model, suggesting that screen-based sedentary behaviour does not necessarily reflect overall diet quality. However, screen time remains linked with less healthy habits, such as higher calorie intake, increased snacking, greater consumption of energy-dense foods and lower levels of physical activity. These factors may be more closely related to variations in body weight and fat accumulation than to adherence to specific dietary patterns.

### 4.4. Limitations and Strengths of the Study

This study has several limitations. First, its cross-sectional design does not allow for causal inference. Second, the relatively small sample size, the pilot nature of the study and its geographical specificity limit the generalizability of the findings, although an expansion of the study to additional municipalities is currently underway. In addition, the absence of an urban comparison group and lack of stratified analyses limit the ability to explore potential differences across settings. The analytical depth is further limited by the absence of analyses of socioeconomic gradients, behavioural clustering and interaction effects, restricting a more comprehensive interpretation of the observed patterns. Future studies involving larger samples and multiple contexts, including urban–rural comparisons, are needed to further validate and expand these findings. Third, reliance on parent-reported information about children may introduce recall bias, potentially affecting the accuracy of dietary and lifestyle data, including information on screen time and physical activity. Moreover, BMI does not differentiate between fat mass and fat-free mass, a well-known limitation of this indicator; nonetheless, this issue is more relevant in clinical assessments than in epidemiological studies, where BMI remains a useful screening tool for monitoring population-level patterns and associations [61]. Among the strengths of this study are the high participation rate, which helped ensure that the sample closely reflected the target population, particularly considering that written consent from both parents was required. In addition, objective measurements of weight and height were collected using standardised procedures by trained personnel, thereby improving the reliability of the anthropometric data. However, participation was voluntary, and the absence of a probabilistic sampling design introduces the possibility of self-selection bias. Families who consented to participate may differ systematically from those who did not, for example, in terms of socioeconomic status, health awareness or engagement with school activities. As a result, the sample cannot be assumed to be fully representative of the target population, and some degree of selection bias cannot be excluded.

## 5. Conclusions

In conclusion, these preliminary data highlight a high prevalence of excess weight, poor diet quality and unhealthy lifestyle behaviours among children in the study area.

Given the pilot nature of the study and its geographical specificity, the findings should be interpreted with caution and may not be generalisable beyond this setting. Further research involving larger samples across multiple rural contexts is warranted to confirm these observations and enhance external validity.

Expanding the study to additional rural municipalities within the Tuscia area may also provide more robust evidence on modifiable determinants, thereby informing the development of targeted, context-specific interventions. From a public health perspective, these findings underscore the need for comprehensive, multisectoral strategies addressing the broader obesogenic environment in which children live, including actions to mitigate the influence of commercial and environmental drivers shaping unhealthy behavioural patterns, while fostering supportive environments that facilitate healthier choices.

Such strategies should also include interventions aimed at improving children’s dietary habits—particularly breakfast quality and daily fruit and vegetable intake—both at school and at home, while also involving parents in nutrition education programs.

Particular attention should be given to rural settings, where structural and contextual barriers may limit access to physical activity opportunities. In this context, strengthening and scaling up school- and community-based initiatives may represent an important component of broader efforts to improve equitable access to physical activity and other health-promoting opportunities.

## Figures and Tables

**Table 1:** Study sample characteristics (mean ± standard deviation and %).

	Girls n = 78 (42.9%)	Boys n = 104 (57.1%)	Total (n = 182)	*p*-Value
Age (years)	8.4 ± 1.4	8.6 ± 1.6	8.5 ± 1.5	* 0.2739
Weight (kg)	29.9 ± 8.0	32.7 ± 9.8	31.5 ± 9.2	* 0.0386
Height (cm)	130.8 ± 9.6	132.7 ± 10.5	131.9 ± 10.1	* 0.2247
BMI (kg/m^2^)	17.2 ± 2.8	18.3 ± 3.5	17.8 ± 3.3	* 0.0254
Ponderal status by IOTF [8]				** 0.3553
Obesity (%)	6.4	10.8	8.9	
Overweight (%)	20.5	27.5	24.4	
Normal weight (%)	61.5	54.9	57.8	
Underweight (%)	11.5	6.9	8.9	
Ponderal status by WHO [42]				** 0.0174
Obesity (%)	6.4	22.5	15.6	
Overweight (%)	24.4	23.5	23.9	
Normal weight (%)	67.9	51.0	58.3	
Underweight (%)	1.3	2.9	2.2	
Mother’s education				
Less than middle school	20.3	22.7	21.7	** 0.5055
High school	47.8	42.3	44.6	
University	27.5	24.7	25.9	
Post degree	4.3	10.3	7.8	
Father’s education				
Less than middle school	25.0	31.0	28.3	** 0.0979
High school	69.1	52.9	60.0	
University	4.4	14.9	10.3	
Post degree	1.5	1.1	1.3	

* Comparison between boys and girls based on *t*-test; ** comparison between boys and girls based on Chi-square test; statistical significance is expressed as *p* < 0.05.

**Table 2:** Food habits and lifestyle variables within the study sample (%).

Variables	% Sample
Mediterranean diet adherence (KIDMED test)	
High	19.9
Average	67.3
Low	12.9
Breakfast habit	
Everyday	70.5
4–6 times/week	11.6
1–3 times/week	12.7
Never	5.2
Fruit consumption	
Several times a day	17.0
One time a day	25.6
4–6 days/week	23.9
1–3 days/week	22.7
Rarely/never	10.8
Vegetable consumption	
Several times a day	9.1
One time a day	17.6
4–6 days/week	19.9
1–3 days/week	31.2
Rarely/never	22.2
Legume consumption	
One time a day	2.3
4–6 days/week	4.7
1–3 days/week	62.8
Rarely/never	30.2
Sugary drinks consumption	
Several times a day	1.7
One time a day	3.5
4–6 days/week	6.4
1–3 days/week	20.2
Rarely/never	68.2
Sweet snacks	
Several times a day	5.8
One time a day	12.1
4–6 days/week	28.9
1–3 days/week	38.7
Rarely/never	14.5
Savory snacks	
One time a day	2.3
4–6 days/week	5.7
1–3 days/week	38.3
Rarely/never	53.7
Sleep time	
≥9 h	80.7
<9 h	19.3
Physical activity unstructured (movement games)	
5/7 days for at least 1 h a day	33.5
<5/7 days for at least 1 h a day	66.5
Physical activity structured	
5/7 days for at least 1 h a day	2.3
<5/7 days for at least 1 h a day	97.7
Physical activity structured and/or movement games	
5/7 days for at least 1 h a day	52.3
<5/7 days for at least 1 h a day	47.7
Screen time (tv/pc/tablet/smartphone/videogames)	
>2 h/day	92.0
≤2 h/day	8.0
Tv in bedroom	
Yes	59.3
No	40.7
Walking to and from school	
Yes	13.1
No	86.8

Data are expressed as n (%).

**Table 3:** Association between selected food habits and lifestyle variables and high Mediterranean diet adherence. Odds ratios (OR) and 95% Confidence Intervals (CIs) from binary logistic regression analysis.

Variables	High Mediterranean Diet Adherence			
	cOR (95% CI)	*p*-Value	aOR (95% CI)	*p*-Value
Sex				
Boys vs. girls	2.69 (1.14–6.35)	0.024 *	1.24 (0.39–3.95)	0.721
Ponderal status by IOFT				
Normal weight/underweight vs. overweight/obese	0.59 (0.27–1.28)	0.180	2.91 (0.59–14.26)	0.187
Ponderal status by WHO				
Normal weight/underweight vs. overweight/obese	0.39 (0.18–0.85)	0.017 *	0.17 (0.04–0.77)	0.021 *
Breakfast habit				
Everyday vs. not everyday	4.84 (1.40–16.71)	0.013 *	5.71 (1.06–30.86)	0.043 *
Fruit consumption				
≥1 time/day vs. <1 time/day	1.95 (0.91–4.16)	0.085	0.49 (0.16–1.52)	0.219
Vegetable consumption				
≥1 time/day vs. <1 time/day	3.72 (1.69–8.18)	0.001 *	3.09 (0.98–9.81)	0.055
Legume consumption				
≥1 time/week vs. <1 time/week	3.92 (1.30–11.81)	0.015 *	2.98 (0.82–10.79)	0.097
Sugary drinks consumption				
Not everyday vs. everyday	1.00 (0.20–4.94)	0.999	0.76 (0.11–5.43)	0.785
Sweet snack				
Not everyday vs. everyday	4.38 (0.99–19.35)	0.052	4.18 (0.56–30.94)	0.162
Screen time (TV, PC, smartphone, videogames)				
≤2 h/day vs. >2 h/day	1.14 (0.30–4.33)	0.851	1.80 (0.35–9.22)	0.483
TV in bedroom				
No vs. yes	1.58 (0.74–3.36)	0.238	1.89 (0.68–5.22)	0.222
Physical activity unstructured				
5/7 days for at least 1 h a day vs. <5/7 days	1.27 (0.58–2.76)	0.553	1.09 (0.42–2.84)	0.855
Sleep time				
≥9 h vs. <9 h	1.93 (0.63–5.92)	0.252	1.83 (0.48–6.93)	0.372
Mother’s education				
Up to high school vs. university/post degree	0.51 (0.23–1.09)	0.083	0.37 (0.14–0.98)	0.045 *

cOR = crude odds ratio; aOR = adjusted odds ratio; CI = confidence interval; variables are mutually adjusted; *p*-value according to Chi-square test; * statistical significance is expressed as *p* < 0.05.

## Data Availability

The datasets presented in this article are not readily available, as the data are part of an ongoing study. Requests to access the datasets should be directed to Dr. Laura Censi (laura.censi@crea.gov.it).

## Supplementary Materials

The following supporting information can be downloaded at: https://www.mdpi.com/article/10.3390/nu18111756/s1, Table S1: KIDMED Test to Assess Adherence to the Mediterranean Diet in Children and Adolescents.
