# Supplementary material for: Ponderal Status, Eating and Lifestyle Habits in Rural School Children: A Pilot Survey of the SBAM-ONFOODS Cohort Study
Source: Nutrients. 2026 May 29;18(11):1756. doi: 10.3390/nu18111756 (PMC13258498; doi:10.3390/nu18111756)
Supplement: Supplementary file 1 [file nutrients-18-01756-s001.zip › nutrients-4302521-supplementary.pdf]

KIDMED Test to Assess Adherence to the Mediterranean Diet in Children and Adolescents

Scoring

+1	Takes a fruit or fruit juice every day
+1	Has a second fruit every day
+1	Has fresh or cooked vegetables regularly once a day
+1	Has fresh or cooked vegetables more than once a day
+1	Consumes fish regularly (at least 2–3 times per week)
-1	Goes more than once a week to a fast-food (hamburger) restaurant
+1	Likes pulses and eats them more than once a week
+1	Consumes pasta or rice almost every day (≥5 times per week)
+1	Has cereals or grains (e.g., bread) for breakfast
+1	Consumes nuts regularly (at least 2–3 times per week)
+1	Uses olive oil at home
-1	Skips breakfast
+1	Has a dairy product for breakfast (e.g., milk, yogurt)
-1	Has commercially baked goods or pastries for breakfast
+1	Takes two yogurts and/or some cheese (~40 g) daily
-1	Takes sweets and candy several times every day

KIDMED = Mediterranean Diet Quality Index for children and adolescents.

Serra-Majem, L.; Ribas, L.; Ngo, J.; et al. Food, youth and the Mediterranean diet in Spain: Development of KIDMED, Mediterranean Diet Quality Index in children and adolescents. Public Health Nutr. 2004, 7, 931–935. https://doi.org/10.1079/PHN2004556.
